# Molecular Mechanisms Underlying the Pain-Relieving Effects of Extracorporeal Shock Wave Therapy: A Focus on Fascia Nociceptors

**DOI:** 10.3390/life12050743

**Published:** 2022-05-17

**Authors:** Larisa Ryskalin, Gabriele Morucci, Gianfranco Natale, Paola Soldani, Marco Gesi

**Affiliations:** 1 Department of Translational Research and New Technologies in Medicine and Surgery, University of Pisa, Via Roma 55, 56126 Pisa, Italy; gabriele.morucci@unipi.it (G.M.); gianfranco.natale@unipi.it (G.N.); paola.soldani@unipi.it (P.S.); marco.gesi@unipi.it (M.G.); 2 Center for Rehabilitative Medicine “Sport and Anatomy”, University of Pisa, 56121 Pisa, Italy; 3 Museum of Human Anatomy “Filippo Civinini”, University of Pisa, 56126 Pisa, Italy

**Keywords:** extracorporeal shock wave therapy (ESWT), fascial innervation, nociceptors, pain relief effect, musculoskeletal disorders, myofascial pain syndrome (MPS)

## Abstract

In recent years, extracorporeal shock wave therapy (ESWT) has received increasing attention for its potential beneficial effects on various bone and soft-tissue pathologies, yielding promising outcomes for pain relief and functional recovery. In fact, ESWT has emerged as an alternative, non-invasive, and safe treatment for the management of numerous musculoskeletal disorders, including myofascial pain syndrome (MPS). In particular, MPS is a common chronic painful condition, accounting for the largest proportion of patients affected by musculoskeletal problems. Remarkably, sensory innervation and nociceptors of the fascial system are emerging to play a pivotal role as pain generators in MPS. At the same time, increasing evidence demonstrates that application of ESWT results in selective loss of sensory unmyelinated nerve fibers, thereby inducing long-lasting analgesia. The findings discussed in the present review are supposed to add novel viewpoints that may further enrich our knowledge on the complex interactions occurring between disorders of the deep fascia including changes in innervation, sensitization of fascial nociceptors, the pathophysiology of chronic musculoskeletal pain of MPS, and EWST-induced analgesia. Moreover, gaining mechanistic insights into the molecular mechanisms of pain-alleviating effects of ESWT may broaden the fields of shock waves clinical practice far beyond the musculoskeletal system or its original application for lithotripsy.

## 1. Introduction

In the last decades, extracorporeal shock wave therapy (ESWT) has emerged as an alternative to surgery, as a non-invasive therapeutic approach for the management of various musculoskeletal disorders affecting both the lumbar region and upper and lower limbs [1].

Being at first introduced in the 1980s into routine clinical practice for treating urinary stones (lithotripsy) [2,3], its application has rapidly expanded across a wide range of medical specialties, yielding promising outcomes for bones and soft tissues healing, pain relief, and functional recovery. Due to the negligible side effects, which only relate to minor referred pain during ESWT sessions and minor hematoma, this therapy represents a safe, advantageous, and well-tolerated approach without surgical risks or severe complications. Therefore, the use of ESWT has gained increasing popularity for treating different musculoskeletal disorders. These encompass tendinopathies (both calcifying and non-calcifying), plantar fasciitis (PF), lateral epicondylitis (“tennis elbow”), greater trochanteric pain syndrome, bone nonunion fractures, and joint diseases including avascular necrosis [4]. In orthopedics, ESWT has demonstrated encouraging results in the treatment of pathological neo-calcifications. Several studies reported a complete or partial disintegration/fragmentation of calcification, pain relief, and a significant improvement of shoulder joint movement in patients with calcifying tendinitis following ESWT [5,6,7,8,9]. Of note, no recurrence of calcium deposits was observed during the two years following ESWT [10]. Moreover, strong evidence supports the use of low-energy ESWT in the treatment of pain-related tendinopathies such as chronic plantar fasciitis [11,12,13].

Although the precise molecular mechanisms of shock waves are still largely unknown, increasing evidence indicates that the application of ESWT to the locomotor system may sort various beneficial effects, which are way beyond a mere mechanical disintegrative effect, as generally assumed. These include increased perfusion, neo-angiogenic effects, osteogenesis, and fibroblastic stimulation [14,15,16,17,18,19]. Moreover, recent publications indicate that ESWT intervention has positive effects in reducing cellular inflammation [20,21]. Experimental findings demonstrate that shock waves modulate macrophage activity, reduce leukocyte infiltration, and regulate cytokine and chemokine production [22,23,24]. Furthermore, shock waves can promote tissue-repairing effects and regeneration by regulating stem cell activities within bones and soft tissues [25,26,27,28].

In addition to the aforementioned cellular and molecular mechanisms induced by shock waves, the pain-relieving effect is the most intriguing one. A number of randomized controlled trials (RCTs) showed that ESWT can effectively relieve pain in chronic PF that has been refractory to other common nonoperative modalities [29,30]. Therefore, currently, there is an increasing interest in ESWT application as a therapeutic alternative for multiple acute and chronic musculoskeletal pain conditions including myofascial pain syndrome (MPS) [31,32,33], defined as a painful disorder characterized by the presence of hyperirritable palpable nodules in the skeletal muscle fibers or the muscle fascia known as myofascial trigger points (MTrPs) [34,35]. MPS, both in acute and chronic form, represents one of the most common disabling musculoskeletal pain syndromes [33,36,37]. Remarkably, referred pain and/or increased pain sensitivity associated with MTrPs constitute the leading criteria to establish a diagnosis of MPS. In addition, dorsal horn sensitization is emerging as a key player in continuous pain of a specific body region as occurring in MPS [38]. Patients affected by MPS suffer from local muscle pain and referred pain originating from the surrounding fascia; this, in turn, reduces the range of motion, inducing muscle weakness and disability, thereby negatively impacting the quality of life. Therefore, pain relief and functional improvement, represent primary endpoints for most of the current therapies, which include both invasive and non-invasive techniques (i.e., dry needling, ultrasound, stretching, massage, taping) [39,40,41,42,43,44]. Nonetheless, it should be noted that most of the prescribed interventions for MPS show limited effectiveness in alleviating pain, which often persists or even worsens over time [45]. By contrast, a growing body of evidence demonstrates that long-term analgesia, lasting between several months and years, occurs following a couple of ESWT sessions [31,35,46,47,48,49,50].

Various hypotheses have been proposed on the molecular mechanisms by which ESWT alleviates pain in the musculoskeletal system [47,48,51,52,53,54]. A hypothesis is that ESWT can alter pain transmission by reducing the release of the pain-related neuropeptide substance P (SP) and other pain mediators from the treated area [52,55]. Furthermore, selective destruction of sensory unmyelinated fibers within the focal zone of shock wave application has shown to play a pivotal role in mediating ESWT-induced long-lasting analgesia [56]. Other authors speculated that dispersion of calcitonin gene-related peptide (CGRP) from degenerated sensory nerve fibers induces neurogenic inflammation preventing local re-innervation [47,48,51].

In this regard, it is worthy of mentioning that compared to the underlying muscles, the fascial tissue shows a greater innervation with multiple sensory nerve fibers including those with nociceptive capability [57,58,59]. Thus, the fascial system is recently, though gradually, emerging as a major contributor in the pathogenesis of musculoskeletal pain and dysfunctions including MPS [58,60,61,62,63,64].

Therefore, in the present review, after providing an overview on the molecular mechanisms underlying the beneficial effects of ESWT on the musculoskeletal system, we discuss the complex relationship between pain in MPS and deep fascia alterations and the possible molecular mechanisms underlying pain-alleviating effects of EWST, with a focus on fascial sensory nerve fibers and pain receptors.

## 2. Physiology and Biology of ESWT

### 2.1. Mechanism of Action: Technical Aspect

ESWT consists of biphasic pulsed acoustic waves generated extracorporeally, which carry energy and propagate in three dimensions through the tissue to induce a rapid increase in pressure [65]. In particular, shock waves consist of rapidly rising positive pressure impulses ranging from 5 MPa up to 120 MPa in 5 ns, followed by a negative pressure of about −20 MPa [66]. Both the positive and negative phases induce physical/mechanical effects on treated tissues (such as absorption, reflection, refraction, cavitation) [21,67], followed by various molecular and biological effects, due to mechano-transduction. In fact, ESWT can trigger the activation of numerous cell signaling pathways, as well as the release of several biomolecules [17,20,21].

### 2.2. Types of ESWT: Focused vs. Radial Extracorporeal Shock Waves

**Figure 1:**
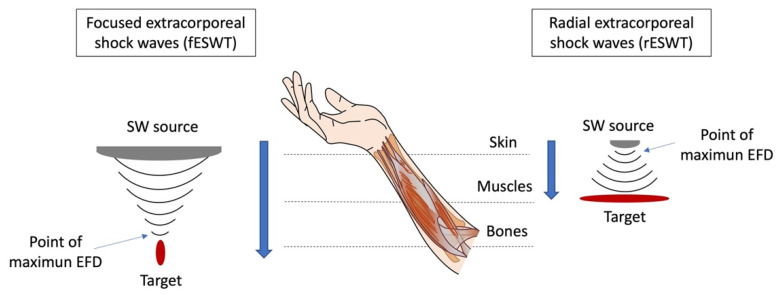
Physical characteristics and wave propagation of fESWT and rESWT. fESWT produces a high-energy shock wave that is concentrated onto a restricted, focused area of the body at a selected depth of the tissue. Thus, this type of shock wave is particularly effective for the deepest tissues (up to 12 cm deep from the surface of the body), where it generates the maximal energy flux density (EFD). By contrast, rSEWT produces a non-focused low-energy shock wave that expands radially from the skin surface. This, in turn, generates a shock wave with the highest pressure at the source, while it gradually weakens as it penetrates deeper into the tissue. Therefore, compared to fESWT, rESWT is generally used to treat wider and more superficial areas of the body.

In clinical practice, two different types of ESWT are used, namely focused shock wave therapy (fESWT) and radial shock wave therapy (rESWT), which differ in the types of devices used for the application. fESWT includes focusing devices (electro-hydraulic, piezo-electric, electro-magnetic flat, electro-magnetic cylindric), while rESWT consists of radial (ballistic) devices. In detail, fESWT produces a focused wave in a small focal area of 2–8 mm diameter, thus generating the maximum of energy flux density (EFD) in an egg-shaped focal zone at a determined depth of the tissue. By contrast, the ballistic source of rESWT results in a non-focused wave, which dissipates radially at the skin, reaching maximal pressure at the source [65]. Thus, focused shock waves (fSW) are concentrated on a restricted area of the body and can penetrate deeper into the tissue, whereas radial shock waves (rSW) are more superficial; thus, rSW are generally used to treat wider areas of the body [21] (Figure 1). Nonetheless, much of current orthopedic research is focused on the application of focused shock waves on the musculoskeletal system [1].

In addition, compared with fESWT generators, rESWT devices generate a maximum pressure that is generally over 100 times lower and a pulse duration of 1000 times longer [68] (Table 1).

EFD is the parameter used in clinical practice by professionals to illustrate the energy flow of shock waves through a treated area with perpendicular orientation to the direction of propagation. The unit of EFD is given in mJ/mm^2^. Thus, ESWT can be classified into low (<0.08 mJ/mm^2^), medium (0.08–0.28 mJ/mm^2^), and high (up to 0.60 mJ/mm^2^) intensities, which may result in different clinical applications [65].

### 2.3. Biological and Molecular Effects of ESWT on Bones and Soft Tissues

In recent years, ESWT has been deeply and extensively studied for multiple biological properties and clinical applications [14,15,16,17,18,19,20,21,22,23,24,25,26,27,28,69,70,71]. There is a large body of literature demonstrating that ESWT application may cause various effects on both bone and soft tissues. For instance, this therapy improves blood supply and thus tissue regeneration at the tendon–bone junction; this occurs through the stimulation of the ingrowth of neovascularization associated with increased expressions of angiogenic and osteogenic growth factors, such as vascular endothelial growth factor (VEGF), endothelial nitric oxide synthase (eNOS), proliferating cell nuclear antigen (PCNA), and bone morphogenetic protein-2 (BMP-2) [15,70]. Chen et al. [72] demonstrated that ESWT treatment can resolve edema, swelling, and inflammatory cell infiltration in injured tendons. Increased expression of transforming growth factor-beta 1 (TGF-β1) and insulin-like growth factor-I (IGF-I) is responsible for initiating ESWT-induced mitogenic and morphogenic responses, which stimulate tenocyte growth, proliferation, and thus tendon repair. An ESWT-induced increase in TGF-β1 can trigger the release of lubricin, a lubricating mucinous glycoprotein that enhances tendon gliding and facilitates tissue healing [73]. Furthermore, shock waves were shown to promote stem cell self-renewal and differentiation, affecting tissue healing and regeneration [25,26,27,28,74]. ESWT promotes osteogenic differentiation of mesenchymal stem cells and bone healing by inducing bone morphogenetic proteins and TGF-β1 [75,76].

Experimental in vitro studies revealed that shock wave treatment can induce various biological effects on different cell types, as a consequence of mechano-transduction. Remarkably, in stimulated cells, ESWT promotes cell proliferation and differentiation, as well as regulation of cell metabolism, migration, and apoptosis [19,20,25,77,78,79]. Moreover, ESWT was shown to trigger the release of several biomolecules such as adenosine triphosphate (ATP) and growth factors and stimulate numerous signaling pathways, thereby affecting gene expression. Examples of cell signaling pathways and molecules involved in the biological response to ESWT stimulation include focal adhesion kinase (FAK), MAPK/ERK ATP/P2X7, PI3K/Akt/mTOR, Wnt/β-catenin, extracellular-signal-regulated kinase (ERK; Erk1/2) and p38 MAPK, toll-like receptor 3 (TLR3), glycogen synthase kinase 3 beta (GSK-3β), protein kinase R-like endoplasmic reticulum kinase/activated transcription factor (PERK/ATF), and so on [21,80].

Besides their application in regenerative medicine, a pain relief effect of shock waves in musculoskeletal disorders has been advocated in current literature. Increasing basic research reports that ESWT can provide analgesic effects through several mechanisms, encompassing degeneration of small sensory unmyelinated nerve fibers, reduction of pain-related neuropeptides, hyperstimulation of nociceptors, and alteration of pain neurotransmission [47,48,51,52,53,54,55].

## 3. A Brief Overview of the Human Fascial System and Its Role as a Pain Generator in Musculoskeletal Dysfunctions

### 3.1. Anatomy and Function of the Fascial and Musculoskeletal System

**Figure 2:**
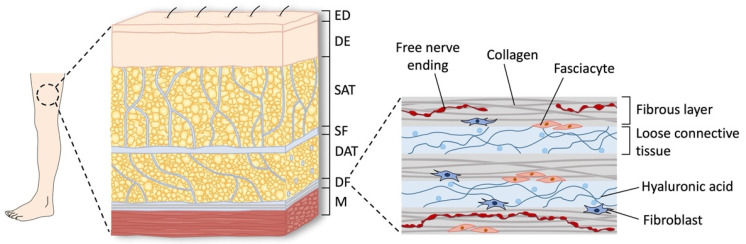
Anatomy of the human fascial system. The figure depicts the pattern of organization of the subcutaneous tissue and the superficial and deep fasciae. At a microscopic level, the DF consists of a multilayered structure of 2–3 fibrous layers of collagen fiber bundles separated by thin layers of loose connective tissue rich in hyaluronic acid (HA). The DF is also enriched in cellular components, namely fibroblasts and fasciacytes. Free nerve endings (FNEs) with several axonal widenings (i.e., varicosities) are also reported. ED = epidermis; DE = dermis; SAT = superficial adipose tissue; SF = superficial fascia; DAT = deep adipose tissue; DF = deep fascia; M = muscle.

The human fascial system is a three-dimensional continuum of soft, collagen-containing, loose, and dense fibrous connective tissue that permeates all of the body and surrounds all muscles, bones, nerve fibers, and organs, thus creating a unique environment for body system functioning [81]. Based on the histological features and anatomical relationships, apart from all of the visceral fasciae, the myofasciae can be classified as superficial and deep fascia [82]. The superficial fascia is a fibro-elastic layer dividing the subcutaneous tissue of the entire body into the superficial adipose tissue (SAT) and the deep adipose tissue (DAT), with a tridimensional network of fibrous septa connecting the skin and the deep fascia. The deep fascia is a fibrous membrane that interpenetrates and envelops all of the muscles of the body (Figure 2).

However, the deep fascia features different characteristics depending on the anatomical region, being either the trunk or the limbs [60,83]. In particular, the trunk deep fascia is composed of a very thin connective tissue layer, while, at the level of the limbs, the deep fascia consists of a multilayered structure consisting of 2–3 layers of parallel collagen fiber bundles. Each layer shows a different orientation of the collagen fibers, thus contributing to the strong resistance to traction of the fascia itself. Moreover, each layer is separated from the adjacent ones by a thin layer of loose connective tissue that allows the various layers to slide over each other. This, in turn, represents a key element of musculoskeletal mobility. In addition, a loose connective tissue rich in hyaluronic acid (HA) occurs between the deep fascia and the underlying epimysium, which again allows the gliding between the muscles and the deep fascia [84,85]. Remarkably, changes in viscoelasticity of the deep fascial system, accumulation of high-molecular-weight (HMW) chains of HA, and altered mobility of fascial adjacent layers have been recently emerging as important causes of fascial nociceptor activation and thus as possible “pain generators” in the MPS [62,64,86,87,88,89].

### 3.2. Sensory Innervation of the Deep Fascia: The Role of Nociceptive Fibers

Although the term “fascia” may sound misleading, this structure is not just an envelope that holds together vessels, nerves, muscles, and organs. Increasing evidence suggests that the fascia is not a passive structure, which merely envelops organs and the whole body, but it rather transmits and receives mechano-metabolic information, thus influencing movement perception, peripheral motor coordination, and proprioception [85,90]. Recent evidence demonstrates an abundant innervation of the deep fascia consisting of both free nerve endings (FNEs), postganglionic sympathetic fibers [91], and various types of encapsulated receptors [92,93,94]. These latter mechanoreceptors may vary from small Golgi–Mazzoni-like bulbs and Ruffini corpuscles up to large Pacinian corpuscles [92,94,95], though other studies found no such corpuscular receptors, neither in animal nor in human samples [91,96,97]. Nonetheless, data were collected from different parts of the thoracolumbar fascia (TLF), either small areas of the fascia lateral to the spinous processes or more lateral areas of the TLF. Specific differences in density innervation and types of fibers occur depending on the fascial area [59,95]. This, in turn, may explain the discrepancies in literature data [91,95].

**Figure 3:**
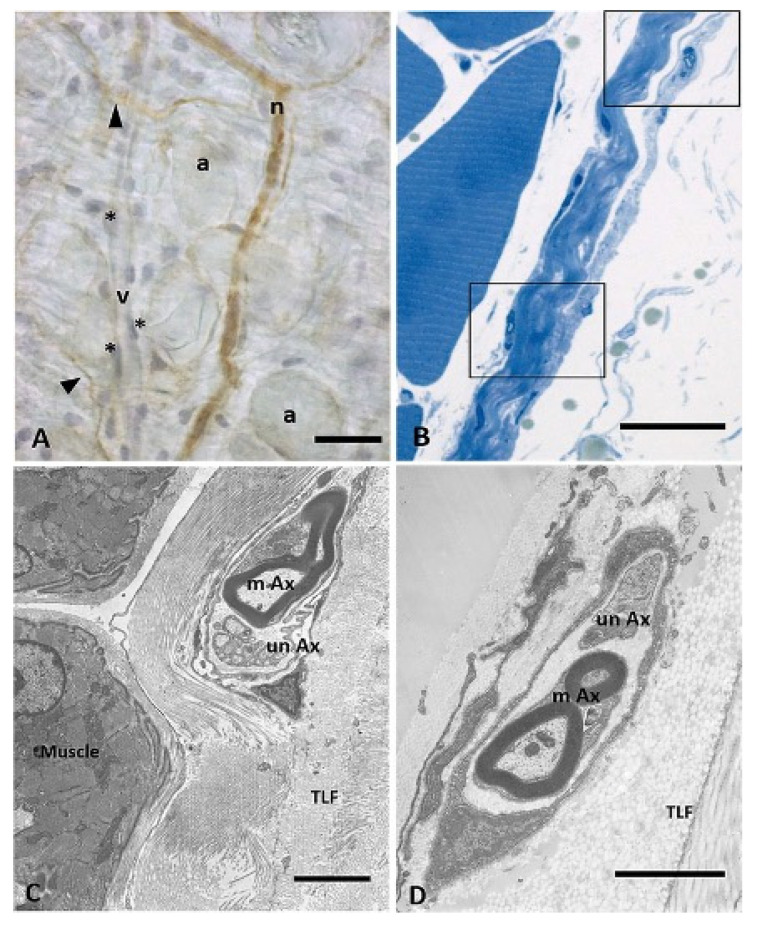
Innervation of mouse fascial system. (**A**) floating TLF stained with anti-S100 antibody and hematoxylin; arrows indicate single nerve fibers (a: adipocytes; n: small nerve; v: vessel; * endothelial cells). (**B**) TLF semithin section; nerve structures in the midst of collagen bundles of the fascial layers are shown in the boxes. (**C**,**D**) TEM images of myelinic (mAx) and unmyelinic (unAx) axons within the inner and outer layers of the TLF, respectively. Reprinted with permission from Ref. [59]. Copyright 2021 Springer Nature.

Beyond typical proprioceptors, such as muscle spindles and Golgi organs, and encapsulated mechanoreceptors, in the last decade evidence on the occurrence of an intrinsic sensory innervation of the deep fascia was provided [57,59,96,97,98,99] (Figure 3).

Remarkably, immunohistochemical investigations demonstrated an abundant innervation of the fascia consisting of nociceptive FNEs [96,97,98,100]. These consist of both thinly myelinated Aδ fibers and small unmyelinated C-fibers [98]. However, differences in conveying specific components of the pain message occur [101]. In detail, Aδ fibers transduce a fast, acute, and well-defined noxious stimulus (“first” or fast pain), whilst C-fibers covey a pain message that is slower, more diffuse, and poorly localized (“second” or slow pain) [101].

**Figure 4:**
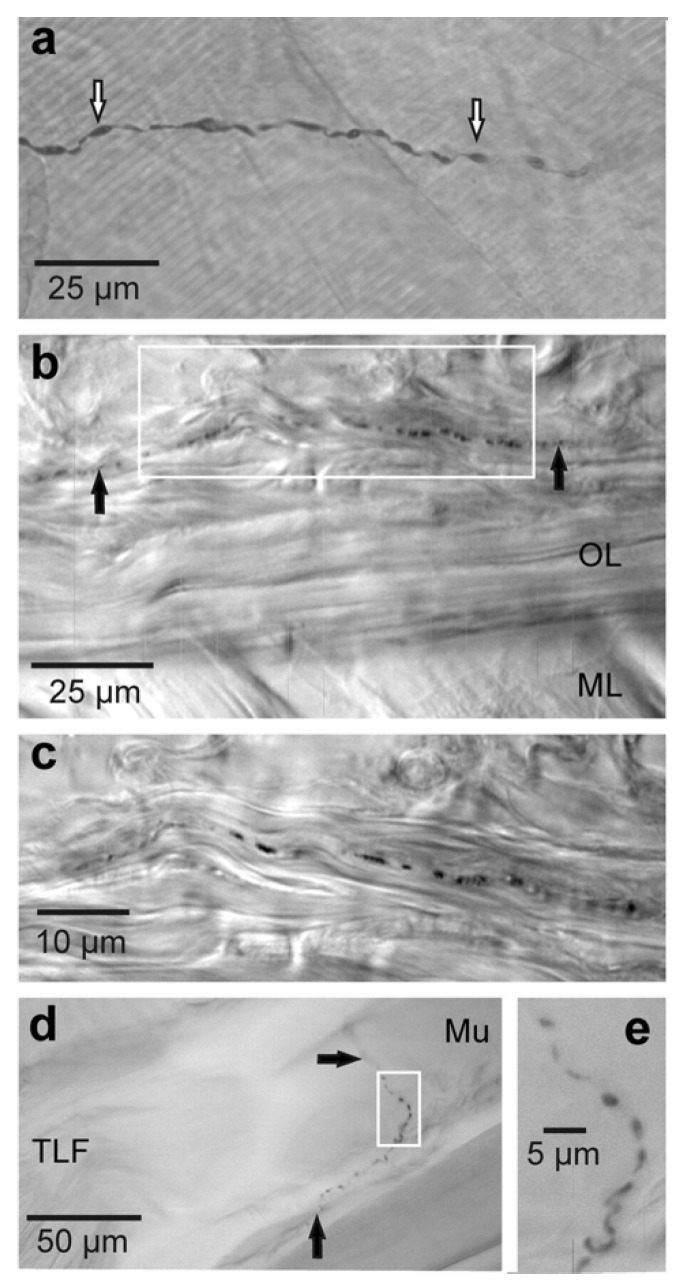
Sensory fibers and nerve endings within the rat TLF. (**a**) CGRP-ir fiber with numerous varicosities (open arrows). (**b**) SP-ir fiber with a chain of varicosities (black arrows) close to the outer layer (OL); ML: middle layer. (**c**) Higher magnification of the area boxed in (**b**). (**d**) SP-ir free nerve ending in a region where cells of a low back muscle (Mu) make contact with collagen fibers of the TLF. (**e**) Higher magnification of the area boxed in (**d**). Reprinted with permission from Ref. [96], Copyright 2011 Elsevier.

**Figure 5:**
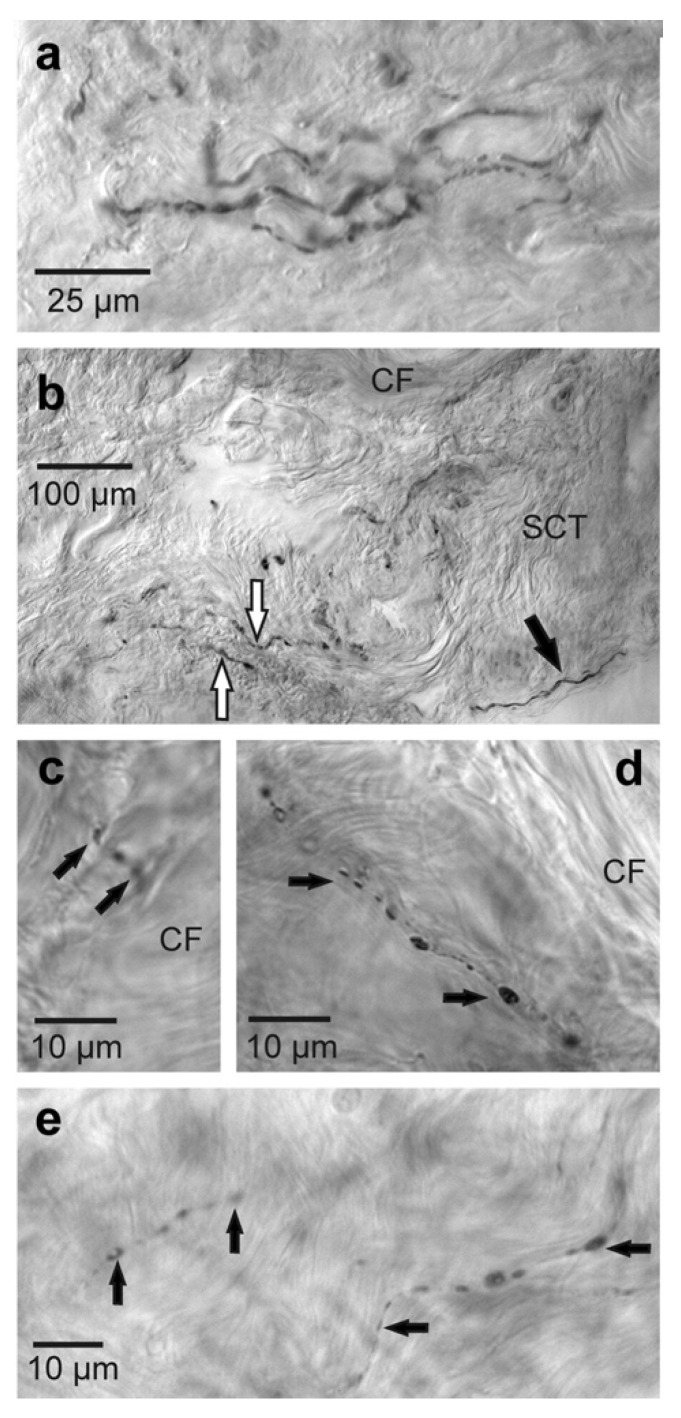
Nerve fibers and endings within the human TLF. (**a**) PGP 9.5-ir nerve fibers between collagen fibers and low back muscle. (**b**) TH-ir fibers on passage (black arrows) and nerve endings (open arrows) within the subcutaneous tissue (SCT) close to TLF collagen fibers (CF). (**c**) SP-ir nerve terminal within the subcutaneous tissue. (**d**,**e**) CGRP-ir free nerve endings with varicosities (arrows). Reprinted with permission from Ref. [96]. Copyright 2011 Elsevier.

At the morphological level, FNEs in the rat (Figure 4) and human (Figure 5) fascia are similar and they appear as a string of pearls with numerous axonal widenings (i.e., varicosities) close to their nerve endings, which contain neuropeptides and neurotrophins [91,96]; in fact, these FNEs are distinguishable from fibers on passage due to the presence of a chain of varicosities, consisting of at least three/four varicosities at the preterminal portion of the axons [91].

Both non-peptidergic and peptidergic axons of unmyelinated C-fibers were found to be densely distributed within the rat crural fascia [98]. Again, Mense and Hoheisel provided histological and functional evidence of nociceptive fibers within the rat TLF [97]; these latter included CGRP- and SP-containing fibers, as well as transient receptor potential cation channel subfamily V member 1 (TRPV1)-immunopositive FNEs [97,99].

Although most of these data were obtained in mice and rats, some findings from the human fascia are also reported in current literature [91,92,94,96]. Numerous small unmyelinated nerves were found in all specimens from different areas of the deep fascia of the upper limbs (without any sign of pathological lesions) obtained from 13 cadavers [95]. In line with this, immunohistochemical analysis against the myelin-forming Schwann cells marker (anti-S100 antibody) revealed a dense network of thin nerve endings within the deep fascia of the human hip [102]. Another recent study performed on twenty formalin embalmed human cadavers documented the occurrence of peripheral nerve endings and nociceptors within the posterior layer of the thoracolumbar fascia (PTLF). Morphometrical analysis demonstrated that these nerve fibers are more abundant in the sacral region than at thoracic vertebral levels of the human PTLF [103].

### 3.3. The Deep Fascia as a Source of Pain in Musculoskeletal Disorders

In recent years, some papers discussed strong morphological and functional evidence for a nociceptive capacity of deep fascia and thus its contribution to various painful musculoskeletal conditions [58,60,61,62,63,64]. Clinical trials on healthy human volunteers demonstrated that electrical high-frequency stimulation of TLF or injection of pain-inducing substances into the TLF evokes long-term potentiation-like pain amplification while generating pain sensations that are more intense and unpleasant when compared to subcutaneous and muscular tissue [58,104,105,106,107].

Compared to the adjacent muscular tissue, the fascial tissue shows a greater and homogeneous distribution of the nerve network [58,59,102]. In particular, sensory nerve fibers with nociceptive capability (i.e., positive for CGRP immunostaining) occurred within the TLF of mice with a density of innervation three times higher than that occurring in back muscles [57]. Thus, it is not surprising that pathological alterations of the fascia may result in intense local and referred pain.

Remarkably, alterations in the degree of innervation of various components of the deep fascial system can occur under pathological conditions [108,109]. For instance, FNE density was significantly increased in the palmar aponeurosis from patients with Dupuytren’s disease compared with normal, non-pathological upper limb fasciae [110].

Furthermore, evidence was provided that increased density of nociceptive fibers occurs in inflamed fasciae, which in turn could explain the pain in several disorders such as fasciitis and non-specific low back pain [89,110,111,112]. Hoheisel et al. reported considerable changes in density innervation in the rat TLF induced by chronic fasciitis [99]. In particular, the authors demonstrated that the density of SP-positive nociceptive fibers was significantly increased within inflamed TLF compared with intact, normal ones, thus suggesting an increased pain sensitivity during fascia inflammation [99]. These effects were accompanied by increased excitation and sensitization of dorsal horn neurons by input from pathologically altered fascia. In fact, chronically inflamed TLF produced a marked expansion of the spinal target region of fascia afferents, extending to the L3 lumbar segment, whilst under normal conditions, this region features ineffective or silent synaptic connections with the TLF [113]. This was confirmed by electrophysiological data showing that pathologically altered (inflamed) TLF induced a marked increase in the proportion of dorsal horn neurons in spinal segment L3, which acquire new inputs from the fascia [113].

In line with these findings, other studies demonstrated that experimentally induced fascia inflammation, which mimics several musculoskeletal painful conditions, can alter the spinal cord processing of nociceptive inputs arising from the TLF nociceptive innervation of the fascia and thus pain perception [97,112,114,115]. In particular, these electrophysiological studies showed that: (i) a considerable percentage (ranging from 6% up to 14%) of dorsal horn neurons in the spinal segments Th13-L2 of naïve rats receive strong inputs from the TLF; (ii) the percentage and responsiveness of dorsal horn neurons is significantly increased in experimentally induced inflamed TLF.

Collectively, all of these observations may denote the deep fascia as a key player in the etiology of pain in different musculoskeletal disorders.

## 4. Possible Molecular Mechanisms Underlying Pain-Relieving Effects of ESWT in the Musculoskeletal System

First reports regarding the potential analgesic effect of ESWT date back to the late 1990s [116,117] (Table 2). A controlled, prospective study reported significant pain alleviation along with functional improvement in patients with chronic “tennis elbow” compared with controls [117]. Several randomized controlled trials (RCTs) were conducted on the potential analgesic effects of ESWT on fascial-related painful musculoskeletal conditions [11,12,31,118,119,120,121,122,123,124,125], yielding promising outcomes. For instance, in patients affected by chronic PF, low-energy shock wave application showed greater pain improvement at six-months follow-up when compared with untreated subjects [126]. ESWT-mediated benefits persisted up to 12 months after the end of treatment [126]. Another study demonstrated a remarkable decrease in visual analog scale (VAS) score and referred pain of focused ESWT on 30 patients with myofascial pain components (myofascial trigger points; MTrPs) at three months after therapy [119].

**Figure 6:**
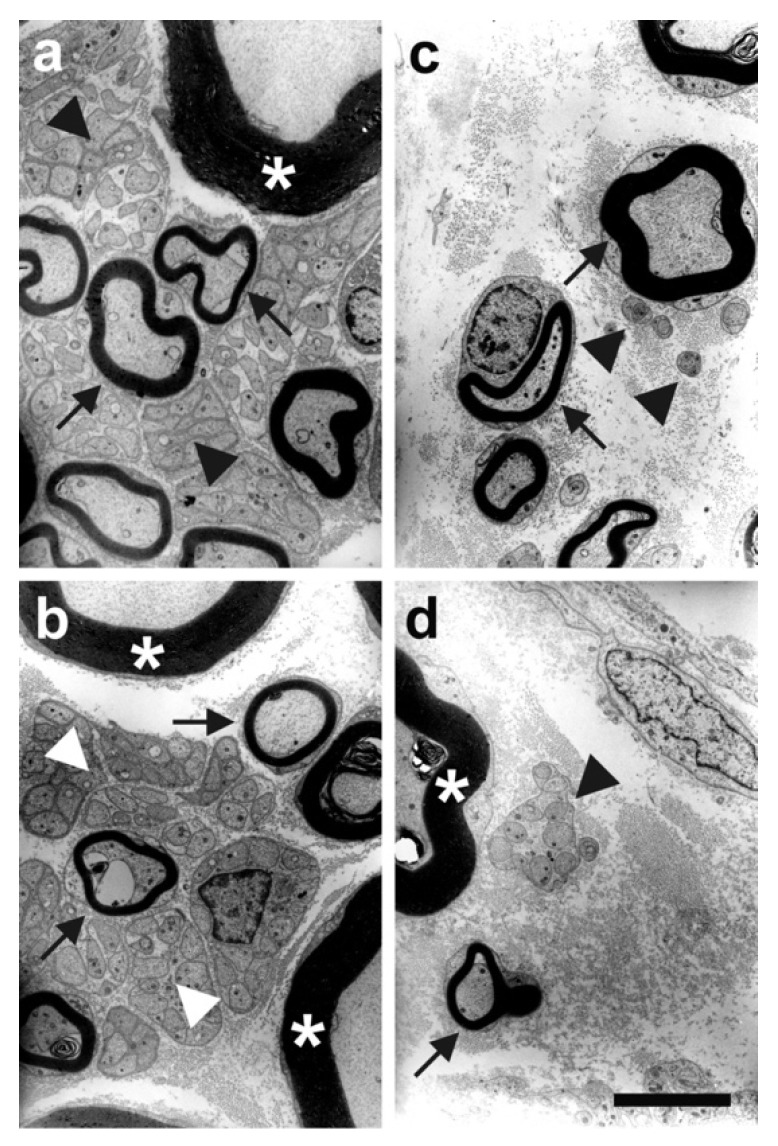
ESWT-induced loss of unmyelinated nerve fibers within the femoral nerve of rabbit hind limb. (**a**) Representative electron microscope images of nerve fibers within the left (**a**,**b**) and right (**c**,**d**) femoral nerve of a rabbit 6 weeks after ESWT application. A substantial reduction of unmyelinated nerve fibers is evident within the ESWT-treated right femoral nerve. Myelin sheet of large myelinated nerve fibers (asterisks); small myelinated nerve fibers (arrows); unmyelinated nerve fibers (arrowheads). Reprinted with permission from Ref. [56]. Copyright 2008 Elsevier.

Although the precise molecular mechanisms underlying ESWT-mediated analgesic effects are not yet fully understood, various mechanisms have been proposed, including: (i) degeneration of small sensory unmyelinated nerve fibers; (ii) loss of SP- and CGRP-containing nerve fibers; (iii) reduction and dispersion of pain mediators, such as SP and other nociceptive metabolites; (iv) hyperstimulation of nociceptors and alteration of pain neurotransmission; (v) increase in local anti-nociceptive mediators [51,52,53,117,127,128,129] (Table 3) (Figure 6).

Although clear evidence on ESWT’s effects on the fascial nociceptive system is still lacking, a mounting body of literature suggests that ESWT may influence the musculoskeletal system by specifically affecting nerve fibers [52,130]. In vivo animal studies demonstrated that the application of ESWT to the musculoskeletal system mediates long-term analgesia resulting from a selective, substantial loss of unmyelinated nerve fibers [53,56] (Figure 6). One study reported myelin nerve sheath separation and nerve disruption following ESWT; this, in turn, resulted in decreased sensory nerve conduction velocity and thus altered peripheral pain perception [130].

**Figure 7:**
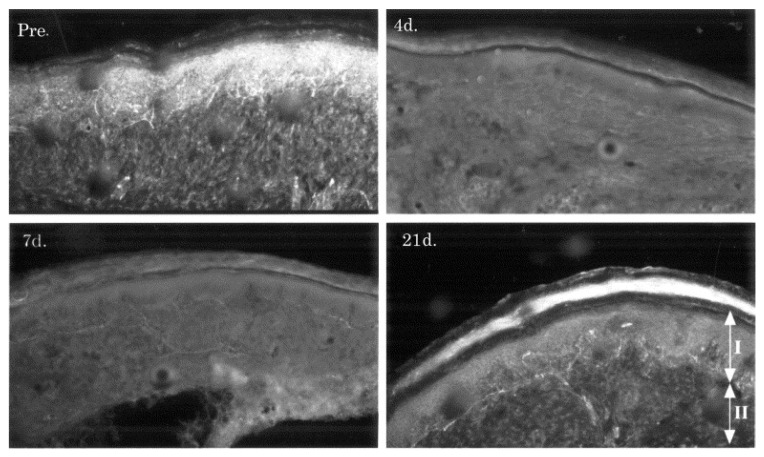
ESWT induces complete degeneration of epidermal nerve fibers in rat hind paw. A significant loss of CGRP-ir nerve fibers was evident in ESWT-treated rat skin at 4, 7, and 21 days following application compared with normal untreated skin. Reprinted with permission from Ref. [51]. Copyright 2001 Elsevier.

Other animal studies showed that a loss of SP in nerve fibers may underly the pain relief effects of EWST [55,129]. EWST exerts anti-nociceptive effects through the reduction of SP-positive neurons in the dorsal root ganglia [132]. In line with this, the application of shock waves decreases both the number of CGRP-immunoreactive (ir) sensory nerve fibers and the percentage of CGPR-ir dorsal root ganglion neurons [51,128,133] (Figure 7).

However, conflicting results have been reported in the literature [134,135,136,137,138]. For instance, Haake and colleagues reported no significant changes in the spinal sensory system or in the expression of SP and CGRP within the dorsal horn of rats after low-energy ESWT [136]. Any modulatory influence on the expression of the spinal opioid system was reported following shock wave application, thus excluding the mechanism of endogenous opioid-related stimulation-produced analgesia for ESWT [135].

Contrariwise, another study revealed that a repetitive application amplified ESWT-induced denervation and pain relief [47]. Repeated exposure (two applications) of shock waves induces longer-lasting degeneration of FNEs, and a longer-acting antinociceptive effect, compared with one single application. An initial application of shock waves caused FNEs degeneration and consequent inflammatory changes, including cytokines synthesis, whereas a second application significantly accentuated inflammatory changes, which prevented reinnervation within the local area [47]. Similarly, three treatments of ESWT, two weeks apart, raised the mechanical nociceptive threshold of the thoracolumbar spine over a 56-day period in horses with clinical evidence of back pain [131].

Furthermore, both gate control concept and hyperstimulation analgesia have been postulated as other possible powerful mechanisms underlying long-lasting analgesic effects of ESWT on the musculoskeletal system [117,134,139]. This latter theory suggests that low-energy ESWT-mediated analgesia may be ascribed to the alteration of nociceptive transmission to the brainstem due to overstimulation of treated areas [127]. Low-energy shock waves may activate small-diameter fibers projecting to the periaqueductal grey (PAG), a key structure of the midbrain in the propagation and modulation of pain [140]; this, in turn, can lessen pain perception through inhibitory serotonergic descending projections at the level of the dorsal horn, thereby exerting an antinociceptive effect [117].

## 5. Conclusions and Future Research Directions

To date, there is growing interest in the application of ESWT on the musculoskeletal system. Mounting evidence demonstrates that ESWT is safe and effective in causing pain reduction and functional recovery in subjects with different musculoskeletal disorders, and especially MPS. Therefore, it is an alternative to surgery.

Among numerous biological effects of ESWT, the pain-relieving effect remains the most intriguing one. Experimental results show that ESWT may influence the musculoskeletal system by specifically affecting nerve fibers. ESWT was shown to induce a selective destruction of sensory unmyelinated fibers, reduction and dispersion of pain mediators and nociceptive metabolites, and prevention of local tissue re-innervation, while altering pain neurotransmission. Moreover, ESWT application reduces the number of CGRP- and SP-positive neurons in the dorsal root ganglia.

Though the mechanisms underlying the etiology of MPS are far from being fully elucidated, a key role of intrinsic sensory innervation and pain receptors of the deep fascia is gradually gaining acceptance. Thus, it seems likely that all of these mechanisms might contribute to the analgetic working mechanism of ESW in MPS by reducing pain sensitivity and dorsal horn sensitization.

However, to date, there is no clear evidence on ESWT effects on the fascial nociceptive system.

Future research efforts should focus on dissecting the ESWT-mediated effects on fascial sensory innervation and nociceptors as well as other molecular mechanisms, which may underlie the long-lasting and dose-dependent analgesic effects of ESWT.

In conclusion, additional knowledge on ESWT modulation of fascial neurological components would impact both the management and treatment of various musculoskeletal disorders, and especially MPS.

## Figures and Tables

**Table 1:** Focused vs. radial shock waves in ESWT.

Physical Characteristics	fSW	rSW
Wave propagation	Focused	Radial
Pressure	Up to 100 MPa	0.1 to 1 MPa
Pulse duration	Does not exceed 2 μs	1 to 5 ms
Penetration power	Up to 10–12 cm	Less than 3 cm
Force of impact	0.001–0.4 mJ/mm EFD^4^ > 0.1 mJ/mm (low-energy shock waves) EFD of 0.2 to 0.4 mJ/mm^2^ (high-energy shock waves)	0.02–0.06 mJ/mm^2^
Energy profile	Rapid rise and fall of the pressure wave	Slower rise and fall of pressure
Target	Skin, muscles, bones	Skin, muscles

Focused shock waves (fSW); radial shock waves (rSW); megaPascal (MPa); energy flux density (EFD).

**Table 2:** Pain-alleviating effects of ESWT: human studies.

Pathological Condition (s)	*n*	Main Finding (s)	Ref.
Chronic proximal PF *	302 patients	Successful result by all four of the evaluation criteria (pain assessment, subject’s self-assessment of pain on first walking in the morning, subject’s self-assessment of activity, use of pain medications) in 56% more of the treated patients 3 months after one treatment	[11]
Chronic PF	112 patients	Decrease in score for pain caused by manual pressure (VAS score from 77 points before treatment to 19 points at 6 months); 25/49 patients able to walk completely without pain	[12]
MPS ^‡^ in upper trapezius	22 patients	Significant decrease in VAS score from 4.91 ± 1.76 to 2.27 ± 1.27; significant increase in pressure threshold from 40.4 ± 9.94 N to 61.2 ± 12.16 N in the treated group	[31]
Painful heel associated with calcaneal spurs	30 patients	Significant alleviation of pain and improvement of function at all follow-ups in treatment group (improvement rating 72.9% at 6 weeks, and 77.4% at 24 weeks)	[116]
Chronic tennis elbow	100 patients	Significant alleviation of pain and improvement of function after treatment, with a good or excellent outcome in 48% and an acceptable result in 42% at 24 weeks	[117]
MTrPs ^#^	30 patients	Significant decrease in VAS score after 3 months’ treatment in 95% of patients (from 3.6 prior to therapy to 1.7 after therapy)	[119]
Carpal tunnel syndrome	36 patients	Significant reduction in VAS and symptom severity score on the Levine Self-assessment Questionnaire at 1 and 3 months after treatment	[120]
Chronic nonspecific low back pain	66 patients	Significant reduction in the VAS score after 2 weeks of therapy (from 6.32 ± 1.12 prior to therapy to 2.96 ± 1.00 after therapy)	[121]
Active myofascial trigger points in the trapezius muscle	64 patients	Statistically significant improvements in the number of trigger points, pain, quality of life, and anxiety scores of patients in both groups (i.e., group I = patients undergoing a single session of low-energy; group II = patients undergoing three sessions of ESWT with the same energy density, with one-week intervals) at 3 and 12 weeks after treatment	[122]
Chronic Low Back Pain	52 patients	An extremely strong analgesic effect in the group treated with rESWT and stabilization training (pain reduction in VAS scale from 4.4 to 2.7 points, on average, at one months and 2.0 points at three months after treatment)	[123]
PF	56 patients	Progressive improvement in the three outcomes evaluated, assessed pain, function, and quality of life (VAS, AOFAS, and SF-36, respectively), at 3, 6, and 12 weeks	[124]
Chronic nonspecific low back pain	140 patients	Significant lower mean numerical rating scale (NRS) values in patients treated with rESWT at 1, 3, and 4 weeks after treatment	[125]
Chronic PF	45 running athletes	Significant reduction of self-reported pain on first walking in the morning, from an average of 6.9 to 2.1 points on a VAS ^†^ score after 6 months; further reduction of pain to an average 1.5 points after 12 months	[126]

* plantar fasciitis; ^†^ visual analog scale; ^‡^ myofascial pain syndrome; ^#^ myofascial trigger points.

**Table 3:** Mechanisms involved in ESWT-induced analgesia: in vivo animal studies.

Animal Model	Site of Application	Effect (s)	**Ref.**
Rat	Foot pad	Amplification of ESWT-induced denervation and pain relief following a repetitive application	[47]
Rat	Plantar skin of hind paw	Nearly complete degeneration of epidermal nerve fibers, as indicated by the significant loss of PGP9.5 and CGRP immunoreactivity	[51]
Rat	Skin of footpads (corresponding to L4 and L5 dermatomes)	Injury of sensory nerve fibers, as indicated by the significant increase in the number of ATF3-ir * DRG ^§^ neurons	[53]
Rabbit	Distal femur	Decrease in substance P release from the periosteum of the femur 6 weeks after ESWT application	[55]
Rabbit	Ventral side of the right distal femur	Selective and substantial loss of unmyelinated nerve fibers within the femoral nerve of treated hind limb	[56]
Rat	Foot pad of hind paw	Reduced CGRP expression in DRG neurons (the percentage of FG ^†^ -labeled CGRP-ir DRG neurons decreased to 18% in treated group)	[128]
Horse	Left forelimb	Significant lower SNCV ^‡^ in treated medial and lateral palmar digital nerves along with a severe disruption of myelin sheath	[130]
Horse	Skin from T12 to L5	Three treatments of ESWT 2 weeks apart raised MNT ^#^ over a 56-day period in horses with back pain	[131]
Rabbit	Ventral side of the right distal femur	Significant decrease in the mean number of SP-ir neurons within DRG L5	[132]
Rat	Hindlimb	Reduction of CGRP-ir DRG neurons innervating the knee in the osteoarthritis model	[133]

* immunoreactive neurons; ^§^ dorsal root ganglion; ^†^ fluorogold crystals (fluorochrome); ^‡^ sensory nerve conduction velocities; ^#^ mechanical nociceptive threshold.

## Data Availability

Not applicable.

## Abbreviations

ATF3	activating transcription factor 3
ATP	adenosine triphosphate
BMP-2	bone morphogenetic protein-2
CGRP	calcitonin gene-related peptide
DAT	deep adipose tissue
EFD	energy flux density
eNOS	endothelial nitric oxide synthase
ERK; Erk1/2	extracellular-signal-regulated kinase
ESWT	extracorporeal shock wave therapy
FAK	focal adhesion kinase
fESWT	focused shock wave therapy
FNEs	free nerve endings
fSW	focused shock waves
GSK-3β	glycogen synthase kinase 3 beta
HA	hyaluronic acid
HMW	high-molecular-weight
IGF-I	insulin-like growth factor-I
MNT	mechanical nociceptive threshold
MPa	megaPascal
MPS	myofascial pain syndrome
MTrPs	myofascial trigger points
PAG	periaqueductal grey
PCNA	proliferating cell nuclear antigen
PERK	protein kinase R-like endoplasmic reticulum kinase
PF	plantar fasciitis
PTLF	posterior layer of the thoracolumbar fascia
RCTs	randomized controlled trials
rESWT	radial shock wave therapy
rSW	radial shock waves
SAT	superficial adipose tissue
SP	substance P
TGF-β1	transforming growth factor-beta 1
TLF	thoracolumbar fascia
TLR3	toll-like receptor 3
TRPV1	transient receptor potential cation channel subfamily V member 1
VAS	visual analog scale
VEGF	vascular endothelial growth factor
